# The Relationship between Cerebral Small Vessel Disease, Hippocampal Volume and Cognitive Functioning in Patients with COPD: An MRI Study

**DOI:** 10.3389/fnagi.2017.00088

**Published:** 2017-03-30

**Authors:** Fiona A. H. M. Cleutjens, Rudolf W. H. M. Ponds, Martijn A. Spruit, Saartje Burgmans, Heidi I. L. Jacobs, Ed H. B. M. Gronenschild, Julie Staals, Frits M. E. Franssen, Jeanette B. Dijkstra, Lowie E. G. W. Vanfleteren, Paul A. Hofman, Emiel F. M. Wouters, Daisy J. A. Janssen

**Affiliations:** ^1^Department of Research and Education, Centre of Expertise for Chronic Organ Failure (CIRO)Horn, Netherlands; ^2^Department of Medical Psychology, Maastricht UMC+/School for Mental Health and Neurosciences (MHeNS)Maastricht, Netherlands; ^3^Department of Respiratory Medicine, Maastricht University Medical Centre, NUTRIM School of Nutrition and Translational Research in MetabolismMaastricht, Netherlands; ^4^Department of Psychiatry and Neuropsychology, School for Mental Health and Neuroscience, Alzheimer Centre Limburg, Maastricht UniversityMaastricht, Netherlands; ^5^Department of Neurology, Maastricht University Medical CentreMaastricht, Netherlands; ^6^Department of Radiology, Maastricht University Medical CentreMaastricht, Netherlands; ^7^Department of Respiratory Medicine, Maastricht UMC+Maastricht, Netherlands

**Keywords:** cerebral small vessel diseases, chronic obstructive pulmonary disease, cognition, hippocampus, magnetic resonance imaging

## Abstract

The neural correlates of cognitive impairment in chronic obstructive pulmonary disease (COPD) are not yet understood. Structural brain abnormalities could possibly be associated with the presence of cognitive impairment through cigarette smoke, inflammation, vascular disease, or hypoxemia in these patients. This study aimed to investigate whether macrostructural brain magnetic resonance imaging (MRI) features of cerebral small vessel disease (SVD) and hippocampal volume (HCV) are related to cognitive performance in patients with COPD. A subgroup of cognitively high and low-performing COPD patients of the COgnitive-PD study, underwent a brain 3T MRI. SVD as a marker of vascular damage was assessed using qualitative visual rating scales. HCV as a marker of neurodegeneration was assessed using the learning embedding for atlas propagation (LEAP) method. Features of SVD and HCV were compared between cognitively high and low-performing individuals using Mann Whitney U tests and independent samples *t*-tests, respectively. No group differences were reported between 25 high-performing (mean age 60.3 (standard deviation [SD] 9.7) years; 40.0% men; forced expiratory volume in first second [FEV_1_] 50.1% predicted) and 30 low-performing patients with COPD (mean age 60.6 (SD 6.8) years; 53.3% men; FEV_1_ 55.6% predicted) regarding demographics, clinical characteristics, comorbidities and the presence of the SVD features and HCV. *To conclude*, the current study does not provide evidence for a relationship between cerebral SVD and HCV and cognitive functioning in patients with COPD. Additional studies will be needed to determine other possible mechanisms of cognitive impairment in patients with COPD, including microstructural brain changes and inflammatory-, hormonal-, metabolic- and (epi)genetic factors.

## Introduction

Chronic obstructive pulmonary disease (COPD) is a highly prevalent chronic disease which is primarily characterized by progressive airflow limitation (Vestbo et al., 2013). Beyond respiratory impairment, patients with COPD often suffer from a variety of comorbid and biological conditions (Divo et al., 2012; Vanfleteren et al., 2013). These biological changes may have adverse effects on the brain leading to cognitive impairment. General cognitive impairment is four times more likely to occur in patients with COPD than in non-COPD controls and involves several cognitive domains, such as psychomotor speed, planning, memory and cognitive flexibility (Cleutjens et al., 2017). Impairments in working and verbal memory have been found in one out of three patients with COPD (Cleutjens et al., 2017). Although the pathogenesis of cognitive impairment in COPD has been searched in oxidative stress, hypoxemia, systemic inflammation and comorbidities, the exact pathways remain unknown (Dodd et al., 2010; Cleutjens et al., 2014a; Lahousse et al., 2015). Structural brain abnormalities are considered an important cause of cognitive impairment, especially decreased hippocampal volume (HCV), which has been associated with decreased memory (Soininen et al., 1994; Schuff et al., 2009; Wolz et al., 2010b), and features of cerebral small vessel disease (SVD; Cai et al., 2015). SVD refers to pathological processes affecting the small arteries, arterioles, venules and capillaries of the brain. For example, chronic hypoperfusion can occur, whereby the supply of oxygen and nutrients to the brain tissue is slowly cut (Cai et al., 2015). Hypoxemia is thought to act in an additive manner in the development of structural brain abnormalities (Miyamoto and Auer, 2000). Structural brain abnormalities associated with SVD can be seen on magnetic resonance imaging (MRI) as white matter hyperintensities (WMHs), lacunes, cerebral microbleeds and perivascular spaces (PVS). The main risk factors for SVD are increased age and hypertension.

In comparison to control participants, COPD patients showed smaller HCV (Li and Fei, 2013), reduced white matter integrity and regional gray matter density (Dodd et al., 2012; Zhang et al., 2012), periventricular white matter lesions (van Dijk et al., 2004), disturbed functional activation of gray matter (Dodd et al., 2012), and a higher prevalence of cerebral microbleeds (Lahousse et al., 2013). Possible mechanisms, common in COPD, which may contribute to brain abnormalities and cognitive impairment are cigarette smoke, inflammation, vascular disease and hypoxemia (Dodd et al., 2010). Yet, the relationship between structural brain abnormalities, especially HCV and features of SVD and cognitive functioning in COPD remains unexplored.

In order to verify whether SVD and HCV may explain the level of cognitive functioning in patients with COPD, this study aimed to assess whether and to what extent SVD and HCV differ between COPD patients with high and low cognitive performance. *A priori*, we hypothesized that COPD patients with low cognitive performances are characterized by a higher SVD load, and smaller HCV compared to COPD patients with high cognitive performances.

## Materials and Methods

### Design

This cross sectional study is part of a longitudinal study (COgnitive-PD study) aimed at mapping and understanding neuropsychological functioning in patients with COPD. Details of the methodology of this study and data concerning cognition have been described before (Cleutjens et al., 2014b, 2017).

### Study Population

For this study, we selected the first 30 cognitively low-performing and the first 25 high-performing patients with COPD during inclusion of the COgnitive-PD study (*n* = 183) who were willing to undergo brain MRI. Six core tests of the detailed neuropsychological test battery from the Cognitive-PD study (Table 1) were used to distinguish cognitively high-performing individuals from low-performing individuals (Burgmans et al., 2009; for details about the tests, please see the Cognitive-PD study protocol (Cleutjens et al., 2014b)). In addition to the exclusion criteria of the COgnitive-PD study (Cleutjens et al., 2014b), patients were not eligible if they had contraindications to undergo a brain MRI (claustrophobia, a cardiac pacemaker, a cochlear implant, a neurostimulator, or other metal implants in the body). The study was approved by the Medical Ethics Committee of the Maastricht University Medical Centre (NL45127.068.13). All patients provided written informed consent to participate in the study.

**Table 1:** **Cognitive performance per core subtest on the COgnitive-PD study neuropsychological test battery**.

	High-performing COPD patients (*n* = 25)		Low-performing COPD patients (*n* = 30)	
Outcome measure	Mean (SD)	*Z* ≤ −1	Mean (SD)	*Z* ≤ −1
VVLT total recall (number correct: 0–75)*	54.4 (8.6)	0.0%	35.4 (9.1)	50.0%
VVLT delayed recall (number correct: 0–15)*	11.3 (2.8)	4.0%	6.3 (2.8)	56.7%
GIT-II animal naming (number of animal names)^‡^	25.5 (5.1)	0.0%	19.2 (5.6)	30.0%
LDST 60 s written (number correct: 0–125)*^§^	33.8 (6.1)	0.0%	23.4 (6.1)	50.0%
CST-C (time in seconds)^†^	32.1 (11.0)	4.0%	50.1 (18.0)	36.7%
SCWT card III (time in seconds)^†^	46.7 (43.4)	16.0%	63.3 (21.9)	80.0%

Abbreviations: CST, Concept Shifting Test; GIT, Groningen Intelligence Test; LDST, Letter Digit Substitution Test; SD, standard deviation; SCWT, Stroop Colour-Word Test; VVLT, Visual Verbal Learning Test; *Z* ≤ −1, percentage of patients with a Z score of −1 or lower. *Higher scores indicate better performance; ^‡^Indicates score has no upper limit; ^§^At least one of out the two LDST 60 subtests had to be scored with a score less than 1.0 SD below the age-, gender- and education-specific mean of the MAAS study population to have an impaired score on the LDST subtest; ^†^Higher scores indicate worse performance.

### Brain MRI Acquisition

Patients had brain MRI on a research-dedicated 3-T MRI scanner (Siemens, Netherlands) operated by research-dedicated technical staff in the Maastricht University Medical Center. Sequences included T1-weighted sagittal sequence (TR = 8.14 ms, TE = 3.72 ms, FA = 8°, FOV = 256 × 256 × 155 mm, acquisition matrix = 256 × 256, number of slices = 155, voxel size = 1.00 mm isotropic), T2-weighted transversal sequence (TR = 3000 ms, TE = 80 ms, FA = 90°, FOV = 230 × 230 × 154 mm, acquisition matrix = 512 × 512, number of slices = 28, slice gap = 0.50 mm, voxel size = 0.45 × 0.45 × 5.5 mm), fluid-attenuated inversion recovery (FLAIR) sagittal sequence (TR = 4800 ms, TE = 275 ms, TI = 1650 ms, FOV = 250 × 250 × 154 mm, acquisition matrix = 240 × 240, number of slices = 275, slice gap = 0.56 mm, voxel size = 1.04 × 1.04 × 0.56 mm), and T2*-weighted transversal sequence (TR = 844 ms, TE = 16 ms, FA = 18°, FOV = 230 × 230 × 154 mm, acquisition matrix = 512 × 512, number of slices = 28, slice gap = 0.50 mm, voxel size = 0.45 × 0.45 × 5.5 mm).

### MRI Analysis

**Figure 1:**
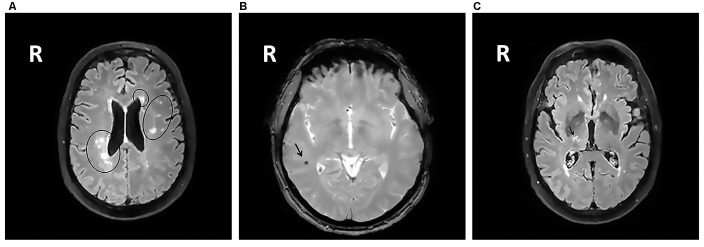
**(A)** Fluid-attenuated inversion recovery (FLAIR) brain magnetic resonance imaging (MRI) scan showing periventricular WMHs and deep WMHs (DWMHs) in both hemispheres. WMH, white matter hyperintensities; R, right. **(B)** T2*-weighted brain MRI scan showing a microbleed located within the right white matter. R, right. **(C)** FLAIR brain MRI scan showing a lacune located in the deep gray matter. R, right.

**Figure 2:**
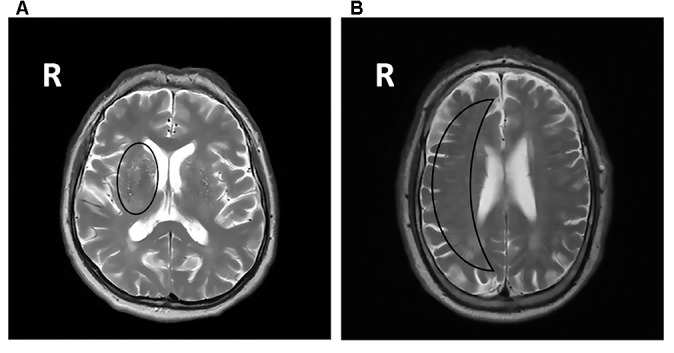
**T2-weighted brain MRI scans showing symmetric extensive PVS at the level of the basal ganglia (A)** and in the centrum semiovale **(B)**. PVS, perivascular spaces; R, right.

All MRIs were analyzed blinded to patient’s cognitive status. WMHs (Figure 1A) were classified as periventricular WMHs (PVWMHs; localized around the lateral ventricles) or deep WMHs (DWMHs; localized within the deep WM) on the Fazekas scale ranging from 0 to 3, using FLAIR and T2-weighted images (Fazekas et al., 1987). Additionally, the age-related white matter changes (ARWMC) scale was applied to rate WMHs more specifically per brain region (Wahlund et al., 2001). PVS (Figure 2) were defined as small, linear or pointy structures of cerebrospinal fluid intensity measuring <3 mm in size and following the course of the vessels, at the level of the basal ganglia and in the centrum semiovale. T2-weighted images were used in order to rate PVS on a qualitative scale (Potter et al., 2015). Microbleeds (Figure 1B) and lacunes (Figure 1C) were rated using T2*, T2 and FLAIR images. Microbleeds were defined as small (<5 mm), homogeneous, round foci of low signal intensity on T2* in the cerebellum, brainstem, basal ganglia, white matter, or cortico-subcortical junction, differentiated from vessel flow voids and mineral depositions in the globi pallidi (Wardlaw et al., 2013a). Lacunes were defined as rounded or ovoid lesions with diameters from 3 mm to 20 mm located in the basal ganglia, internal capsule, centrum semiovale, or brainstem and carefully distinguished from WMH and PVS (Wardlaw et al., 2013b). An SVD compound score, expressing the level of cerebral SVD load, was calculated according to the method of Staals et al. (2014) WMHs and PVS were rated by two trained raters. In a random sample of one third of the brain scans, the agreement between raters was high with intraclass correlation coefficient (ICC) greater than 0.80 for the Fazekas scale (both PVWMH and DWMH); ICC greater than 0.75 for the ARWMC except for the basal ganglia and the infratentorial area; ICC greater than 0.80 regarding PVS in the basal ganglia and greater than 0.65 in the centrum semiovale. Microbleeds and lacunes, were rated by an experienced neurologist (JS). Since one single MRI scanner performed all measurements no intervariability study was performed. HCV was measured on T1-weighted images using the Learning Embeddings Atlas Propagation (LEAP) method (Wolz et al., 2010a). HCV was normalized by multiplying the native space volume with the affine scaling factor (ASF), also derived with LEAP. An ASF value of >1 indicated expansion and a value <1 contraction required to register each individual’s brain to the atlas template (Buckner et al., 2004). LEAP data were provided by Wolz (Ixico Ltd. and Imperial College, London).

### Other Measures

Age, gender, educational level (according to The Dutch Standard Classification of Education (C.B.S., 2001)), marital status, visual and hearing impairment, handedness, smoking behavior and self-reported hypertension and comorbidities (Charlson Comorbidity Index (Charlson et al., 1987)) were recorded. Moreover, data on body mass index (BMI), long-term oxygen therapy, modified Medical Research Council (mMRC) dyspnea scale (American Thoracic Society, 1982), post-bronchodilator spirometry (forced vital capacity (FVC), FVC% predicted, forced expiratory volume in the first second (FEV_1_), FEV_1_% predicted and FEV_1_/FVC), resting arterial blood gases (arterial oxygen partial pressure [PaO_2_], arterial partial pressure of carbon dioxide [PaCO_2_] and oxygen saturation [SaO_2_]), and single breath carbon monoxide diffusing capacity (DLCO% predicted) were collected.

### Statistical Analyses

All statistical analyses were done using SPSS 21.0 (SPSS Inc. Chicago, IL, USA). A *p*-value < 0.05 was considered as statistically significant. Categorical variables are described as frequencies, while continuous variables were tested for normality and are presented as mean and standard deviation (SD) or median and interquartile range (IQR).

Raw test scores on the neuropsychological test battery were transformed into age, gender and education corrected *Z*-scores based on normative data from the Maastricht Aging Study (MAAS). Cognitively low-performing individuals had a score less than 1.0 SD below the age-, gender- and education-specific mean of the MAAS study population for at least two core tests and high-performing individuals had a score more than 1.0 SD above the age-, gender- and education-specific mean of the MAAS study population for at least two core tests (Table 1; Singh et al., 2013).

Patient characteristics were compared between the groups using Chi-square tests for categorical variables and independent samples *t*-test or Mann-Whitney U test, as appropriate for continuous variables. Mann Whitney U tests were performed in order to compare high and low-performing individuals based on features of SVD. HCV were compared using independent samples *t*-tests. The *p*-values were adjusted using the Benjamini-Hochberg correction (Benjamini and Hochberg, 1995) for multiple tests, with a false discovery rate of 5%.

## Results

### Patient Characteristics

Cognitively low-performing patients had significantly worse mean scores on all core tests of the COgnitive-PD test battery compared to high-performing patients (Table 1). Demographic and clinical characteristics, including smoking behavior, FEV_1_, arterial blood gases and diffusion capacity were comparable between cognitively high and low-performing patients. The groups did not differ on reported comorbidities (Table 2).

**Table 2:** **Characteristics of the study population**.

Characteristic	High-performing COPD patients (*n* = 25)	Low-performing COPD patients (*n* = 30)	Benjamini-Hochberg *P*-value
**Demographics**			
Age, years	60.3 (9.7)	60.6 (6.8)	0.941
Male, *n* (%)	10 (40.0)	16 (53.3)	0.497
Lower education, *n* (%)	8 (32.0)	7 (23.3)	0.476
**Exacerbation history in the previous 12 months**
0–1 exacerbations, *n* (%)	10 (40.0)	10 (33.3)	0.408
2 ≥ exacerbations, *n* (%)	15 (60.0)	20 (66.7)	
**Health condition**			
Visual impairment, *n* (%)	4 (16.0)	7 (23.3)	0.524
Hearing impairment, *n* (%)	6 (24.0)	6 (20.0)	0.612
BMI (kg/m^2^), mean, (SD)	25.1 (5.2)	27.5 (7.7)	0.458
Oxygen therapy, *n* (%)	6 (24.0)	3 (10.0)	0.458
mMRC (grade), mean (SD)	2.1 (2.0)	2.6 (2.0)	0.458
**Smoking behavior**			
Current smoker, *n* (%)	3 (12.0)	7 (23.3)	0.458
Former smoker, *n* (%)	20 (80.0)	23 (76.7)	
Never smoker, *n* (%)	2 (8.0)	0 (0.0)	
**Spirometry and arterial blood gases**
FEV_1_/FVC, mean (SD)	37.7 (13.5)	47.0 (14.6)	0.272
FEV_1_ (% predicted), mean (SD)	50.1 (20.1)	55.6 (20.0)	0.497
SaO_2_ (%), mean (SD)	93.7 (2.7)*	94.0 (2.6)	0.844
SaO_2_ before 6MWT (%), mean (SD)	94.5 (2.5)	94.3 (2.1)	0.771
		88.3 (6.5)	
SaO_2_ after 6MWT (%), mean (SD)	87.8 (7.1)	9.6 (1.5)	0.770
	9.3 (1.4)*		
PaO_2_ (kPa), mean (SD)	9.3 (1.1)^‡^	9.7 (1.6)^§^	0.458
			0.361
PaO_2_ off-oxygen (kPa), mean (SD)	5.8 (1.8)*	6.4 (2.2)	
PaCO_2_ (kPa), mean (SD)	5.8 (2.0)^‡^	6.2 (2.1)^§^	0.497
		49.6 (17.4)	0.534
PaCO_2_ off-oxygen (kPa), mean (SD)	49.7 (18.6)*		
DLCO (% predicted), mean (SD)			0.988
**Comorbidities**			
Charlson comorbidity index score, mean, (SD)	2.6 (1.6)	3.5 (2.0)	0.425
Myocardial infarction, *n* (%)	2 (8.0)	10 (33.3)	0.272
Congestive heart failure, *n* (%)	2 (8.0)	5 (16.7)	0.497
Peripheral vascular disease, *n* (%)	4 (16.0)	9 (30.0)	0.458
Cerebrovascular disease, *n* (%)	3 (12.0)	6 (20.0)	0.497
Connective tissue disease, *n* (%)	6 (24.0)	8 (26.7)	0.631
Peptic ulcer disease, *n* (%)	4 (16.0)	4 (13.3)	0.631
Mild, moderate or severe liver disease, *n* (%)	2 (8.0)	0 (0.0)	0.458
Diabetes mellitus, *n* (%)	3 (12.0)	8 (26.7)	0.458
Hemiplegia, *n* (%)	2 (8.0)	2 (6.7)	0.705
Moderate to severe chronic kidney disease, *n* (%)	1 (4.0)	1 (3.3)	0.775
Solid or malignant tumors, *n* (%)	3 (12.0)	6 (20.0)	0.497
Obstructive sleep apnea, *n* (%)	3 (12.0)	7 (23.3)	0.476
Hypertension, *n* (%)	2 (8.0)	11 (36.7)	0.272
HADS anxiety score	6.9 (4.7)	9.1 (4.2)	0.425
HADS anxiety >10 points, *n* (%)	5 (20.0)	11 (36.7)	0.458
HADS depression score (points)	7.0 (4.6)	7.9 (3.5)	0.553
HADS depression >10 points, *n* (%)	5 (20.0)	8 (26.7)	0.523

Abbreviations: 6MWT, six-minute walking test; BMI, body mass index; COPD, chronic obstructive pulmonary disease; FEV_1_, forced expiratory volume in first second; FVC, forced vital capacity; FEV_1_/FVC, Tiffeneau index; HADS, Hospital Anxiety Depression Scale; IQR, interquartile range; PaCO_2_, arterial partial pressure of carbon dioxide; PaO_2_, arterial oxygen partial pressure; SaO_2_, oxygen saturation; SD, standard deviation. *n = 24; ^‡^*n* = 18; ^§^*n* = 27.

### Structural Brain Abnormalities in High and Low-Performing Patients with COPD

No differences were seen on the DWMH and PVWMH Fazekas subscales (Table 3). WMHs mapped specifically per brain region did not differ between low and high-performing patients. PVS in basal ganglia or centrum semiovale did not differ between the groups. There was no difference in presence of lacunes or microbleeds. The level of cereberal SVD load was comparable between the two groups. No significant difference was found between low and high-performing individuals regarding the left and right HCV (Table 3).

**Table 3:** **SVD and (normalized) hippocampal volume**.

		High-performing COPD patients (*n* = 25)	Low-performing COPD patients (*n* = 30)	Benjamini-Hochberg *P*-value
**SVD**				
**WMH Fazekas scale**			
PVH, median (IQR)		1.0 (0.0–1.5)	1.0 (0.0–2.0)	0.509
DWMH, median (IQR)		1.0 (1.0–1.0)	1.0 (1.0–2.0)	0.329
**WMH ARWMC total**, median (IQR)		4.0 (4.0–6.0)	6.0 (3.8–10.0)	0.329
Frontal, median (IQR)	Left	1.0 (0.5–1.0)	1.0 (1.0–1.0)	0.329
	Right	1.0 (1.0–1.0)	1.0 (1.0–1.0)	0.702
Parietal occipital, median (IQR)	Left	1.0 (0.5–1.0)	1.0 (1.0–2.0)	0.329
	Right	1.0 (1.0–1.0)	1.0 (1.0–2.0)	0.386
Temporal, median (IQR)	Left	0.0 (0.0–0.5)	0.0 (0.0–1.0)	0.329
	Right	0.0 (0.0–1.0)	0.0 (0.0–1.0)	0.509
Basal ganglia, median (IQR)	Left	0.0 (0.0–0.0)	0.0 (0.0–0.0)	0.643
	Right	0.0 (0.0–0.0)	0.0 (0.0–0.0)	0.509
Infratentorial, median (IQR)	Left	0.0 (0.0–0.0)	0.0 (0.0–1.0)	0.329
	Right	0.0 (0.0–0.0)	0.0 (0.0–1.0)	0.329
**PVS**			
Basal ganglia, median (IQR)		0.0 (0.0–1.0)	0.0 (0.0–1.0)	0.985
Centrum semiovale, median (IQR)		1.0 (0.0–1.0)	1.0 (0.0–2.0)	0.329
**Lacunes**, *n* (%)		5 (20.0)	5 (17.0)	0.792
**Microbleeds**, *n* (%)		2 (8.0)	1 (3.3)	0.603
**Total SVD score**, median (IQR)		1.0 (0.0–2.0)	0.0 (0.0–2.0)	0.761
**Hippocampal volume**			
Left HCV (mm^3^), mean (SD)		2813.7 (328.1)	2648.8 (328.2)	0.329
Right HCV (mm^3^), mean (SD)		2834.3 (264.6)	2751.2 (243.0)	0.418

*Abbreviations: ARWMC, the age-related white matter changes scale; COPD, chronic obstructive pulmonary disease; DWMH, deep white matter hyperintensity; HCV, hippocampal volume; PVS, enlarged perivascular spaces; IQR, interquartile range; PVH, periventricular hyperintensity; SD, standard deviation; SVD, small vessel disease*.

## Discussion

### Key Findings

The goal of the current brain MRI study was to assess whether differences in SVD features and/or HCV are related to the level of cognitive functioning in patients with COPD. In contrast to our hypothesis that COPD patients with low cognitive performances are characterized by a higher SVD load, and smaller HCV compared to COPD patients with high cognitive performances, we found that structural brain changes were comparable between COPD patients with low or high cognitively performance.

We did not find a difference in the compound score of SVD between high and low-performing COPD patients. Indeed, SVD may also be found in individuals with normal cognitive performance (Duning et al., 2005) and the exact pathophysiological link between SVD features and cognitive impairment remains not completely understood. It is suggested that individuals with normal cognitive performance have a greater cognitive reserve by which they are able to compensate certain brain pathologies better than others (Stern et al., 2005). However, the low and high-performing groups in our study were comparable for educational level, which is considered to be one on of the best indicators for cognitive reserve. Although not significant, we observed a trend towards more hypertension, one of the main risk factors for SVD (Pantoni and Garcia, 1997), in cognitively low-performing patients. 34.5% of patients without physician-diagnosed hypertension had an elevated systolic blood pressure (≥140) or elevated diastolic blood pressure of ≥90. Yet, the percentage of patients with elevated systolic or diastolic blood pressure did not differ between the low and high-performing group.

When we mapped WMHs specifically per brain region, these structural brain abnormalities were not able to differentiate both groups. A population-based study reported that COPD patients had more severe PVWMHs than persons without COPD (van Dijk et al., 2004). Characteristics of the control group were not reported. Our results show no difference in DWMHs between low and high-performing patients. Contradictory findings on the differential impact of PVWMHs and DWMHs on cognition exist, probably due to varying terminology and definitions for PVWMHs and DWMHs (Kim et al., 2008). Soriano-Raya et al. (2012) suggested that only DWMHs, not PVWMHs, are related to cognitive impairments in middle-aged individuals. De Groot et al. (2000) showed that PVWMHs are associated with cognition and DWMHs with depression. High and low cognitively performing COPD patients did not differ on symptoms of depression.

Van Dijk et al. (2004) did not find an association between COPD and lacunes. In addition, our study showed no difference in the incidence of lacunes between high and low-performing COPD patients. In a large population-based study, a higher prevalence of cerebral microbleeds was associated with COPD (Lahousse et al., 2013). Our study did not find differences in the presence of microbleeds between high and low-performing COPD patients. It might be that patients with COPD are at risk for microbleeds due to comorbid processes, such as systemic inflammation (Maclay and MacNee, 2013) without specific cognitive consequences.

Finally, no differences in the extent of PVS were found between high and low-performing patients. The exact causes of PVS are uncertain but abnormalities at the blood brain interface and inflammation have been associated with PVS (Wuerfel et al., 2008; Wardlaw et al., 2009). Studies suggest that smoking and reduced lung function cause inflammation and that both variables might act in an additive manner (Gan et al., 2005; Pelegrino et al., 2013). Our groups were comparable for lung function and smoking status which might explain the absence of differences in the extent of PVS.

MRI markers of SVD including WMHs, lacunes and microbleeds have been related to cognitive performance in population-based (Prins et al., 2005) and stroke cohorts (Gregoire et al., 2011; Patel et al., 2013). Yet, effect sizes have been small across studies (van der Vlies et al., 2012; Kloppenborg et al., 2014). The relatively weak or inconsistent correlations may be explained by the fact that it is not the individual lesions that determine the impact on cognitive performance, but the cumulative effect of these small, spatially distributed lesions. This is in line with findings of Schneider et al. (2007) who demonstrated multiple brain pathologies in cases with dementia using autopsy.

Rates of hippocampal atrophy are sensitive features of neurodegeneration. In normal aging (Ystad et al., 2009) and in several neurological disorders, including AD (Laakso et al., 1995), temporal lobe epilepsy (Kilpatrick et al., 1997), and depressed elderly (Steffens et al., 2011), measurements of volumes of the hippocampus already have been shown to be positively correlated with impaired performances on neuropsychological tests, especially in the memory domain. Also in COPD, HCV may be a proximity marker for cognitive (memory) impairment. Yet, no differences were observed in left and right HCV between cognitively low and high-performing COPD patients. Indeed, compared to cross-sectional studies, effect sizes are stronger across longitudinal studies (Raz et al., 2007).

Alternate mechanisms than structural brain abnormalities may explain cognitive performances are probably multifactorial. First, worse cognitive performance in the low-performing COPD group might be explained by damage caused by oxidative stress, like oxidized proteins, glycated products and lipid peroxidation which lead to degeneration of neurons and impairments in cognitive functioning (Popa-Wagner et al., 2013). Second, although not significant, the high-performing group more often received supplemental oxygen. Regular use of supplemental oxygen therapy has been shown to decrease the risk for cognitive impairment in patients with COPD (Thakur et al., 2010). Third, it is possible that the low-performing group had more comorbidities associated with cognition, other than mentioned in the Charlson comorbidity index (e.g., osteoporosis and Major Depressive Disorder) compared to the high-performing group. Fourth, mitochondrial dysfunction in COPD affects tissues with high energetic demands such as skeletal muscle, cardiac muscle and the central nervous system accompanied by cognitive deficits (Mancuso et al., 2009).

### Limitations

Although we used a 3-T MRI scanner, the prevalence of detected SVD features was rather low. Furthermore, the number of patients investigated in this study was small and therefore we might have a low power to detect group differences in the outcome of interest. However, the group was relatively homogeneous with regard to demographical and clinical characteristics. Nevertheless, selection bias cannot be excluded, because, for instance, patients with personal interest are more willing to participate. We used visual rating scales, and although validated, these have some limitations, such as non-linearity of data, lack of sensitivity to small changes, and subjective assessment. By using non-parametric tests and calculating an ICC, we tried to compensate for these limitations. Moreover, *Z* scores below −1.0 SD and above +1 SD were considered as low and high cognitive performance, respectively. These score cutoff scores may not have been able to differentiate both groups sufficiently. Yet, Singh et al. (2013) considered a *Z* scores below −1.0 SD as being impaired in a COPD population. Though not the goal of this study, future inclusion of a control group could provide more insight into COPD specific brain-cognition correlations. Additionally, this cross-sectional study can only determine associations, no causal relationships nor the sequence of the development of cognitive impairment. Therefore, future studies should assess cognitive impairment at various stages of the disease, taking into account varying degrees of hypoxemia.

### Conclusions

Using macrostructural MRI features of SVD and HCV, we found no differences between cognitively low and high-performing patients with COPD. This suggests that SVD and HCV are not related to cognitive performance in patients with COPD. Additional studies will be needed to get a better understanding of the mechanisms leading to cognitive impairment in COPD patients, including inflammatory factors, hormonal responses, metabolic disturbances, (epi)genetic or lifestyle factors, or microstructural brain changes using Diffusion Tensor Imaging.

## Author Contributions

FAHMC, RWHMP, MAS, SB, HILJ, EHBMG, JS, FMEF, JBD, LEGWV, PAH, EFMW and DJAJ: substantial contributions to the conception or design of the work, and/or the acquisition, analysis or interpretation of data for the work; drafting the work or revising it critically for important intellectual content; final approval of the version to be published.

## Funding

This work was supported by The Weijerhorst Foundation, Maastricht, Netherlands.

## Conflict of Interest Statement

The authors declare that the research was conducted in the absence of any commercial or financial relationships that could be construed as a potential conflict of interest.
